# Supplementary material for: Oral Immunization of Larvae and Juvenile of Lumpfish (Cyclopterus lumpus) against Vibrio anguillarum Does Not Influence Systemic Immunity
Source: Vaccines (Basel). 2021 Jul 23;9(8):819. doi: 10.3390/vaccines9080819 (PMC8402551; doi:10.3390/vaccines9080819)
Supplement: Supplementary file 1 [file vaccines-09-00819-s001.zip › Supplemetary files, Dang et al/Suplmentary Figures S1-S7.pdf]

vaccines

Supplementary figures

Oral Immunization of Larvae and Juvenile of Lumpfish (*Cyclopterus lumpus*) against *Vibrio anguillarum* Does Not Influence Systemic Immunity

My Dang [1], Trung Cao [1], Ignacio Vasquez [1], Ahmed Hossain [1], Hajarooba Gnanagobal [1], Surendra Kumar [2], Jennifer R. Hall [3], Jennifer Monk [4], Danny Boyce [4], Jillian Westcott [5] and Javier Santander [1,*]

[1] Marine Microbial Pathogenesis and Vaccinology Laboratory, Department of Ocean Sciences, Memorial University of Newfoundland, St. John's, A1C 5S7, NL, Canada; tmtdang@mun.ca (M.D.); ttcao@mun.ca (T.C.); ivasquezsoli@mun.ca (I.V.); ahossain@mun.ca (A.H.); hgnanagobal@mun.ca (H.G.)

[2] Ocean Frontier Institute, Department of Ocean Sciences, Memorial University of Newfoundland, St. John's, A1C 5S7, NL, Canada; surendrak@mun.ca

[3] Aquatic Research Cluster, CREAIT Network, Department of Ocean Sciences, Memorial University of Newfoundland, St. John's, A1C 5S7, NL, Canada; jrhall@mun.ca

[4] Dr. Joe Brown Aquatic Research Building (JBARB), Memorial University of Newfoundland, St. John's, A1C 5S7, NL, Canada; jmonk@mun.ca (J.M.); dboyce@mun.ca (D.B.)

[5] Fisheries and Marine Institute of Memorial University of Newfoundland, St. John's, A1C 5S7, NL, Canada; jillian.westcott@mi.mun.ca

* Correspondence: jsantander@mun.ca; Tel.: +1-709-8643268

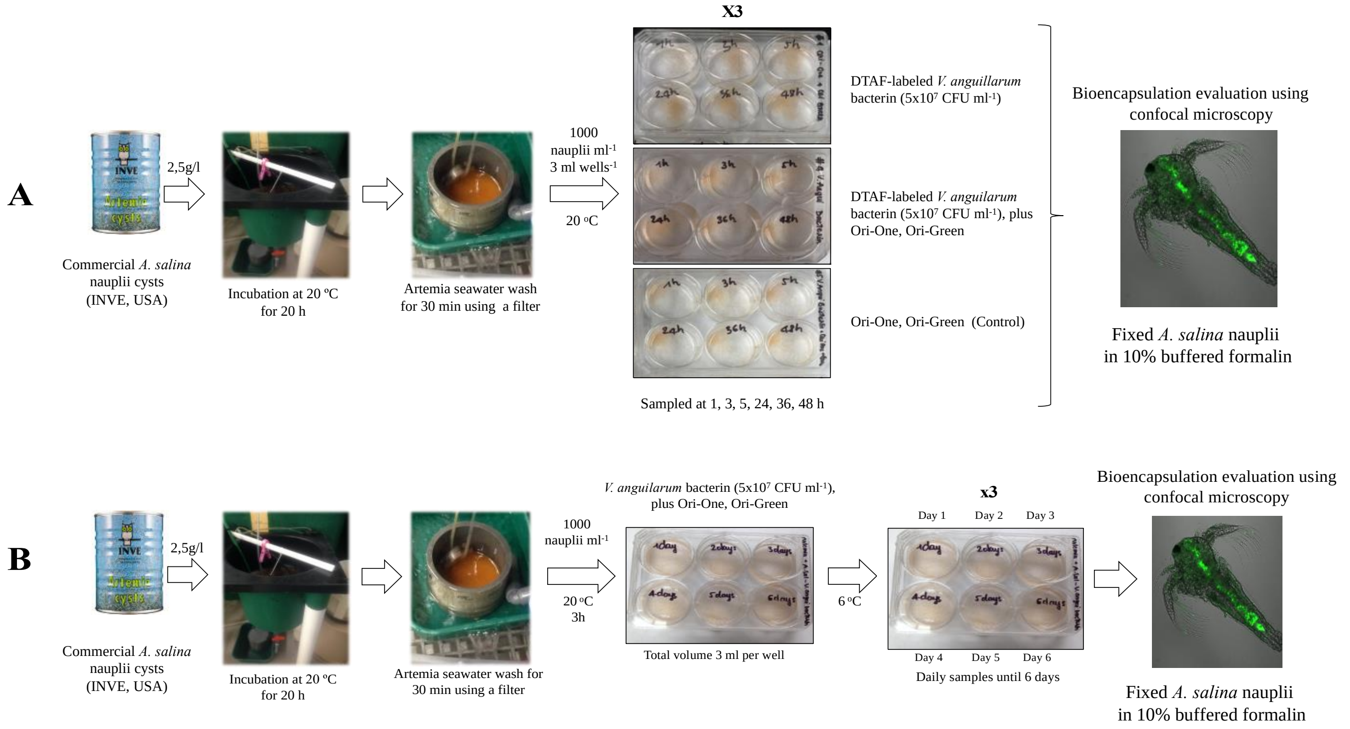

Figure S1. Experimental design for optimization of *V. anguillarum* bacterin bio-encapsulation in *A. salina* nauplii. (A) Optimization of bio-encapsulation conditions and time. The effects of supplementation with Ori-One and Ori-Green on *V. anguillarum* bacterin bio-encapsulation in *A. salina* were assessed. In addition, to determine the optimal incubation time for bio-encapsulation to occur, incubations were performed at 20 ºC for either 1, 3, 5, 24, 36 or 48 h post-inoculation. The presence of the *V. anguillarum* bacterin in the *A. salina* intestine was then assessed using confocal microscopy. **(B) The stability of the *V. anguillarum* bacterin in the intestine of *A. salina* nauplii post bio-encapsulation.** Once the bio-encapsulation process was completed under the optimal conditions determined in (A), *A. salina* containing the *V. anguillarum* bacterin were stored at 6 ºC. The presence of the *V. anguillarum* bacterin in the *A. salina* intestine was then assessed daily for 6 days using confocal microscopy.

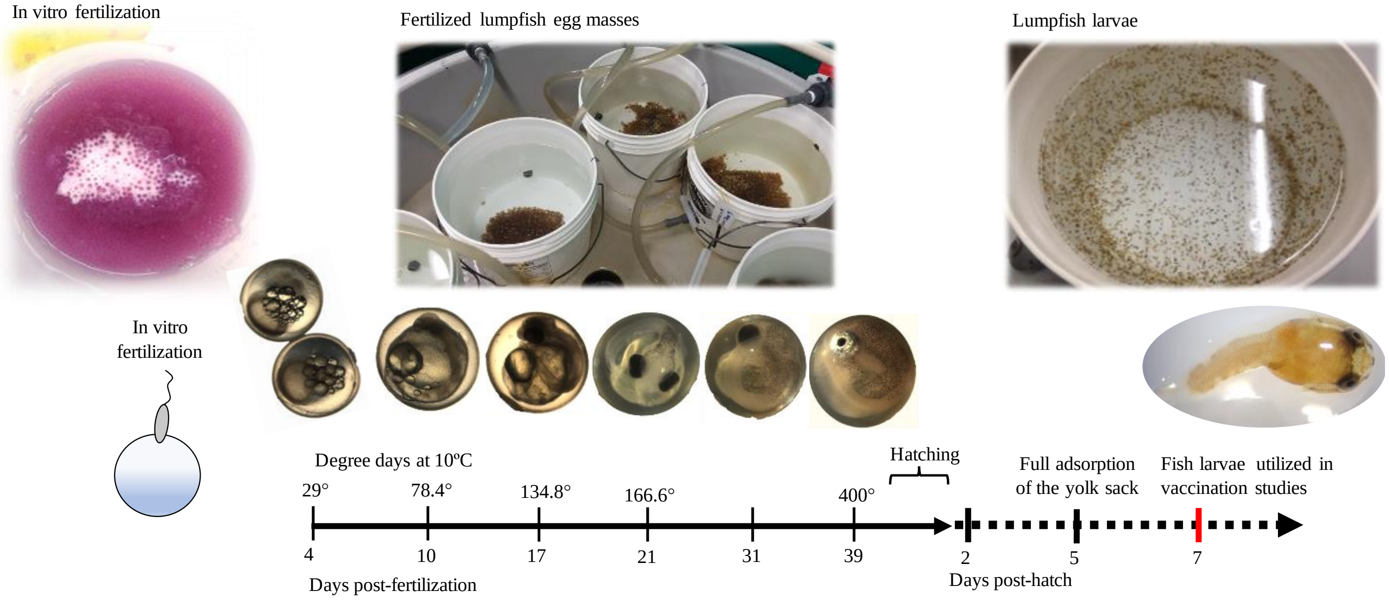

Figure S2. Lumpfish culture conditions and embryonic development. Fertilized lumpfish egg masses were maintained in 5 l buckets (see section 1.7 for details) during embryonic development: **(A)** Segmentation and compression of yolk lipids**;** **(B)** Embryo with oocysts and more developed eyes; **(C)** Eye pigmentation and otoliths**;** **(D)** Skin pigmentation**;** **(E)** The embryo's body**;** **(F)** Large embryo ready to hatch. At 7 dph, the larvae were subjected to vaccination studies.

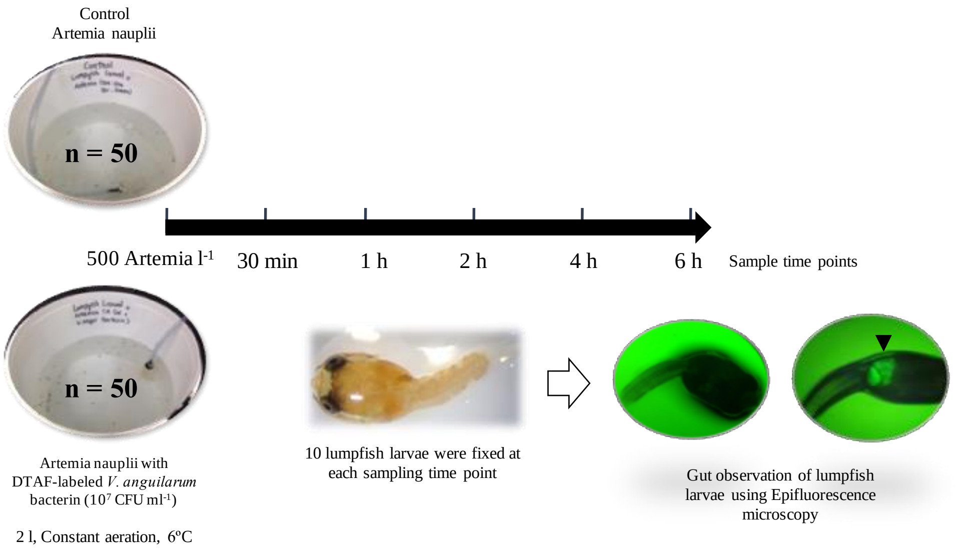

Figure S3. Oral immunization of lumpfish larvae with the DTAF-labeled *V. anguillarum* bacterin bio-encapsulated in *Artemia* nauplii. Lumpfish larvae (7 dph) were either fed *Artemia* nauplii with the bio-encapsulated DTAF-labeled *V. anguillarum* bacterin or control *Artemia* nauplii that had been inoculated with seawater, and maintained at 6 °C for 12 h. The presence of *V. anguillarum* bacterin in the gut of lumpfish larvae compared to non-orally immunized fish was then assessed at 0, 0.5, 1, 2, 4, and 6 h post-oral immunization by fluorescence microscopy. The arrow indicates the presence of green-fluorescent *V. anguillarum* bacterin.

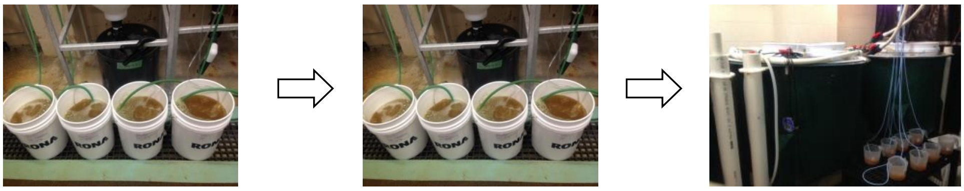

Figure S4. *V. anguillarum* bacterin bio-encapsulation in *A. salina* nauplii for industrial application.

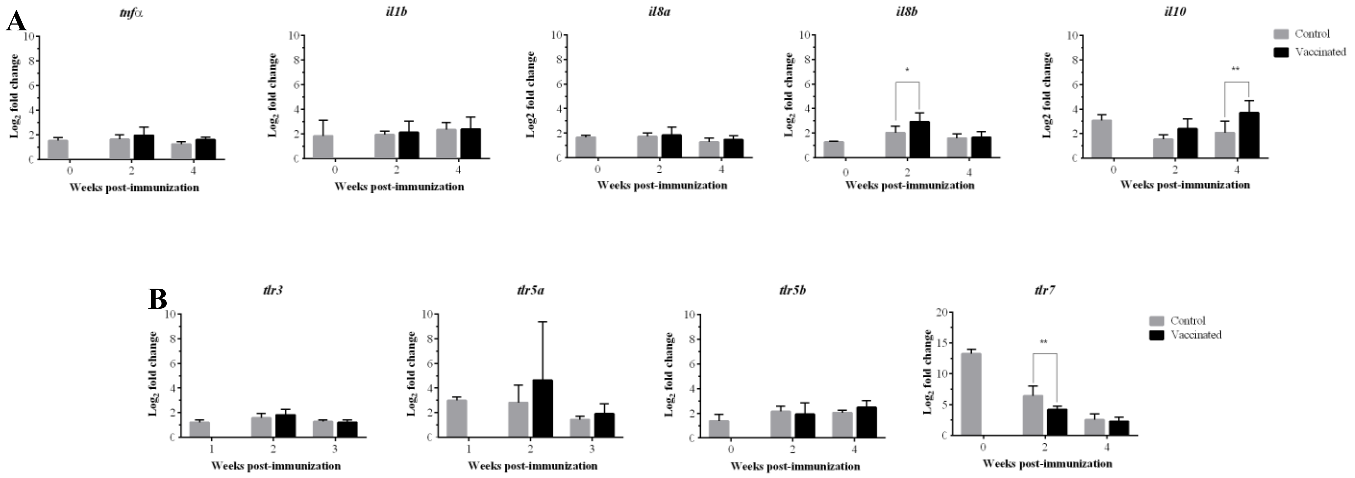

Figure S5. Transcript expression levels of cytokines (A) and toll-like receptors (B) in lumpfish larvae orally immunized with the *V. anguillarum* bacterin bio-encapsulated in *A. salina* nauplii. Transcript expression levels were assessed pre-immunization (n= 3 pools of 10 larvae each), and at 2 weeks (n= 3 pools of 10 larvae each) and 4 weeks (n= 3 pools of 5 larvae each) post-immunization in both mock-orally (control) and orally immunized (vaccinated) larvae. Relative expression was calculated using the RQ values. A two-way ANOVA test, followed by Sidak multiple comparisons post hoc test was used to assess significant differences between the treatments (control and vaccinated) at each time point. Asterisks (*) represent significant differences (*p< 0.05, **p< 0.01, ***p< 0.001).

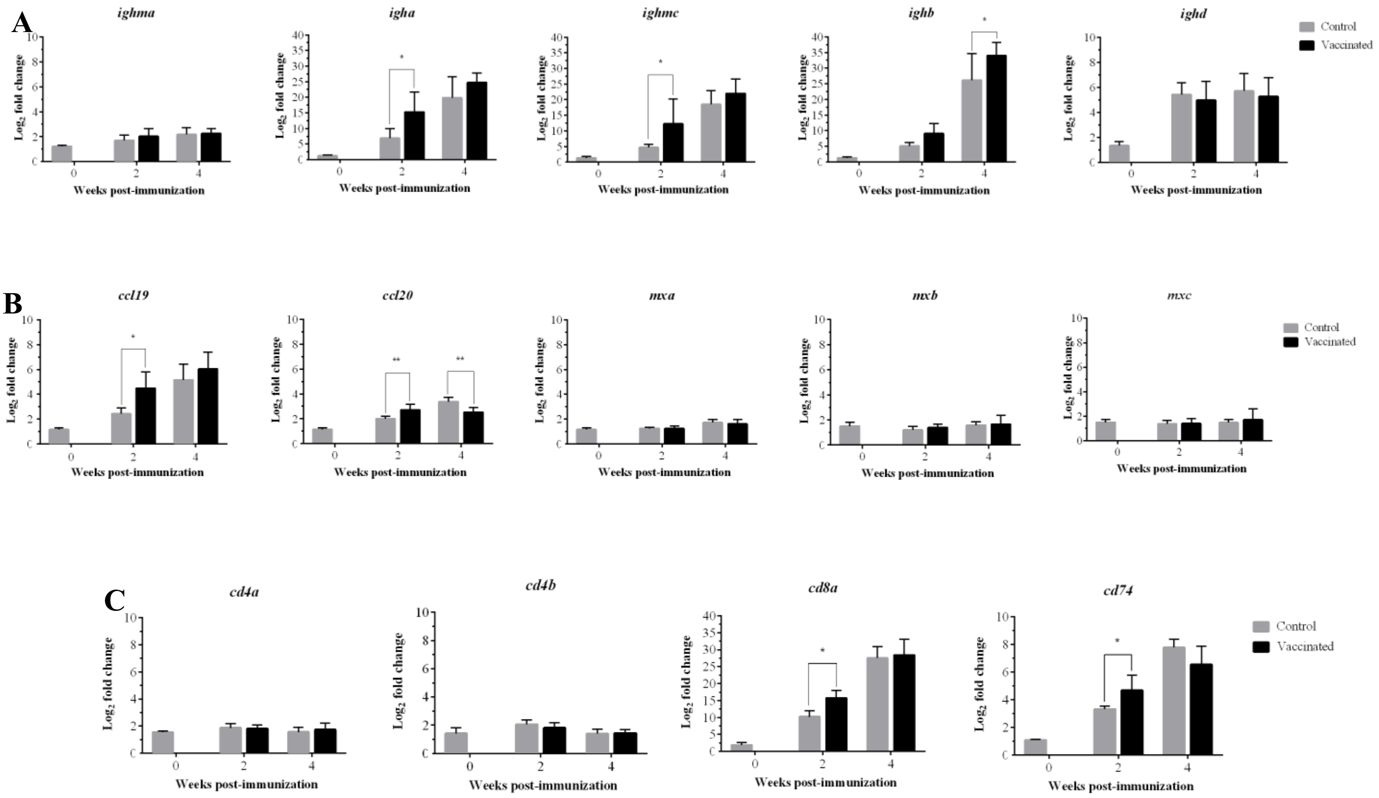

Figure S6. Transcript expression levels of immunoglobulin heavy locus genes (A), cytokine CC genes and interferon-induced GTP-binding proteins (B) and cluster of differentiation genes (C) in lumpfish larvae orally immunized with the *V. anguillarum* bacterin bio-encapsulated in *A. salina* nauplii. Transcript expression levels were assessed pre-immunization (n= 3 pools of 10 larvae each), and at 2 weeks (n= 3 pools of 10 larvae each) and 4 weeks (n= 3 pools of 5 larvae each) post-immunization in both mock-orally (control) and orally immunized (vaccinated) larvae. Relative expression was calculated using the RQ values. A two-way ANOVA test, followed by Sidak multiple comparisons post hoc test was used to assess significant differences between the treatments (control and vaccinated) at each time point. Asterisks (*) represent significant differences (*$p < 0.05$, **$p < 0.01$, ***$p < 0.001$).

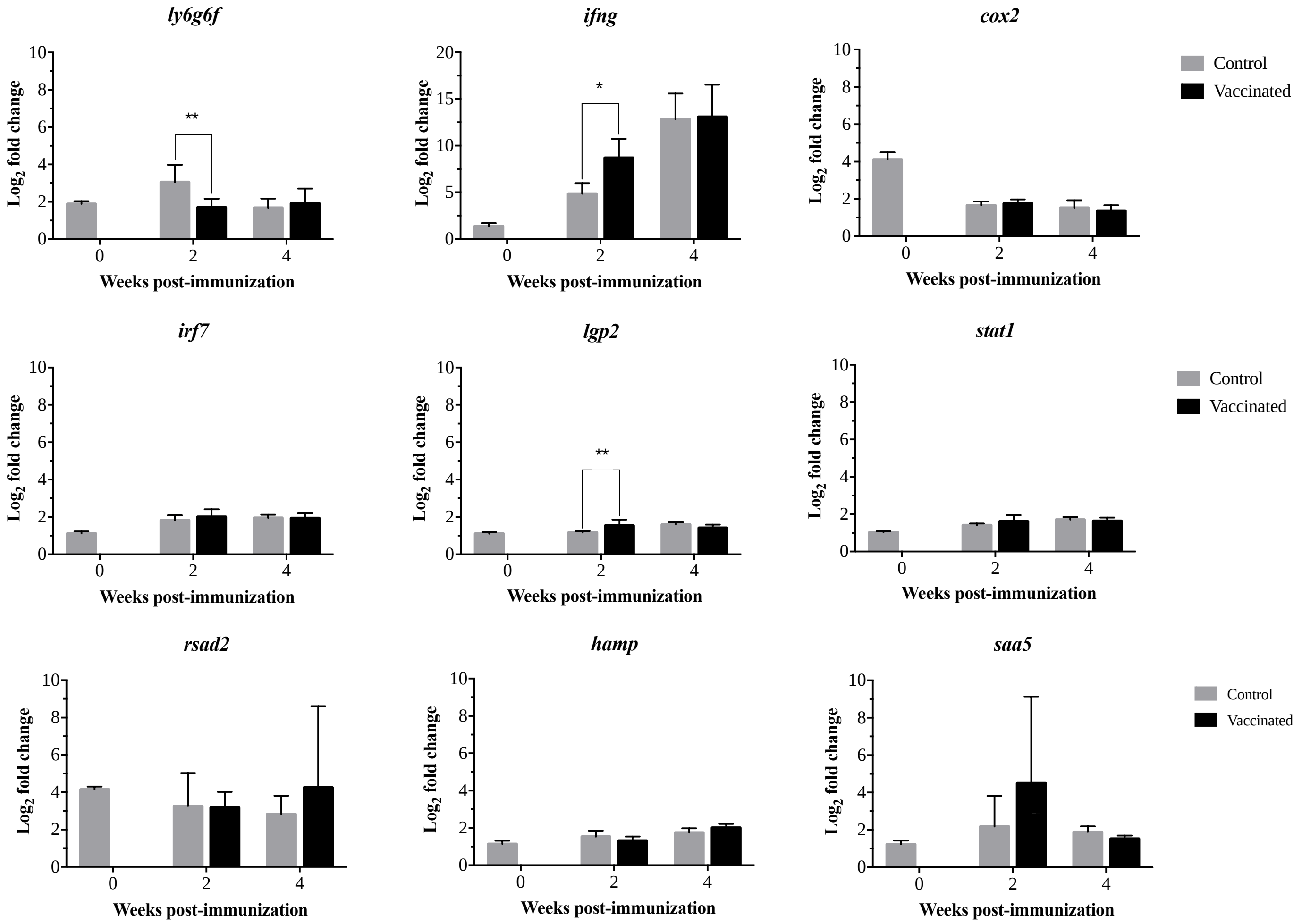

Figure S7. Transcript expression levels of other immune-related genes in lumpfish larvae orally immunized with the *V. anguillarum* bacterin bio-encapsulated in *A. salina* nauplii. Transcript expression levels were assessed (n= 3 pools of 10 larvae each), and at 2 weeks (n= 3 pools of 10 larvae each) and 4 weeks (n= 3 pools of 5 larvae each) post-immunization in both mock-orally (control) and orally immunized (vaccinated) larvae. Relative expression was calculated using the RQ values. A two-way ANOVA test, followed by Sidak multiple comparisons post hoc test was used to assess significant differences between the treatments (control and vaccinated) at each time point. Asterisks (*) represent significant differences (*$p < 0.05$, **$p < 0.01$, ***$p < 0.001$).
