# Supplementary material for: Oral Immunization of Larvae and Juvenile of Lumpfish (Cyclopterus lumpus) against Vibrio anguillarum Does Not Influence Systemic Immunity
Source: Vaccines (Basel). 2021 Jul 23;9(8):819. doi: 10.3390/vaccines9080819 (PMC8402551; doi:10.3390/vaccines9080819)
Supplement: Supplementary file 1 [file vaccines-09-00819-s001.zip › Supplemetary files, Dang et al/Supplementary Table S1.pdf]

**Table S1. Sequences of primer pairs used in qPCR analyses of transcript expression levels in lumpfish (*Cyclopterus lumpus*) larvae.**

| Gene name (symbol)[a] | Trinity ID from NCBI SRA acc. no. SRP238224 | Nucleotide sequence (5'-3') | Amplicon Size (bp) | Efficiency (%) |
|---|---|---|---|---|
| C-C motif chemokine-like 19 (*ccl19*) | DN10492_c0_g1_i4 | F: GCTCAGGTACCAACGGACTG<br>R: CGTGTCCTCCGATCTGTCTC | 94 | 94.33 |
| cyclooxygenase-2 (*cox2*) | DN750_c1_g1_i1 | F: GAATTCCTCACCTGGGTCAA<br>R: ATGGCATCTCTGAGGAAGGA | 122 | 99.18 |
| hepcidin anti-microbial peptide (*hamp*) | DN2993_c0_g1_i4 | F: GCTCGCCTTTATTTGCATTC<br>R: ATATGCCGCAACTGGAGTGT | 100 | 93.36 |
| interleukin 8_a (*il8a*) | DN21169_c0_g1_i2 | F: AAGTCATAGCCGGACTGTCG<br>R: CCCTGCTGATGGAGTTGTCT | 109 | 100.39 |
| interleukin 8_b (*il8b*) | DN4613_c0_g1_i4 | F: GTCTGAGAAGCCTGGGAGTG<br>R: TCAGAGTGGCAATGATCTCG | 138 | 98.15 |
| interleukin 10 (*il10*) | DN41536_c0_g1_i1 | F: AACCAGTGCTGTCGTTTCGT<br>R: TGTCCAAGTCATCGTTTGCT | 106 | 95.24 |
| ATP-dependent RNA helicase lgp2 (*lgp2*) | DN49186_c0_g1_i1 | F: GCAACCTGGTGGTACGCTAT<br>R: CTCGGCGACCACTGAATACT | 104 | 81.54 |
| interferon-induced GTP-binding protein_a (*mxa*) | DN526_c0_g1_i6 | F: TGCACAGACTCAAGCAGAGC<br>R: CCACACTTGAGCTCCTCTCC | 144 | 85.43 |
| interferon-induced GTP-binding protein_b (*mxb*) | DN526_c0_g1_i3 | F: TTGCGGCTTGGAAAAATATC<br>R: TCCACGGTACCTTCGTTCAT | 95 | 92.78 |
| interferon-induced GTP-binding protein_c (*mxc*) | DN237_c1_g1_i1 | F: GGAAGTGGCAGACATTGTGA<br>R: CTGCTGCAATCTCCTTCTCC | 131 | 90.70 |
| signal transducer and activator of transcription 1 (*stat1*) | DN3250_c2_g1_i2 | F: CTCAAGATGCTGGACTGCAA<br>R: ATGCTCTCGATCCACTTGCT | 104 | 84.99 |
| toll-like receptor 3 (*tlr3*) | DN30532_c0_g1_i1 | F: AGAGGGCAGGGAATTTGAGT<br>R: TGCACGAGTCATTCTCCAAG | 101 | 90.29 |

| | | | | |
|---|---|---|---|---|
| C-C motif chemokine-like 20 (*ccl20*) | DN9266_c0_g1_i3 | F: ATGGGCTACACCATCCAGAC<br>R: CCACTTGGATGAAGGGTCAG | 102 | 80.07 |
| immunoglobulin heavy chain variable region a (*igha*) | DN1665_c0_g3_i2 | F: AGGACTGGAGTGGATTGGAA<br>R: TGCATGGTCTGTCCGTTTAG | 129 | 91.00 |
| immunoglobulin heavy chain_b (*ighb*) | DN1665_c0_g4_i1 | F: GAATGGAACAAGGGGACAAA<br>R: CGGTCGTTGAGTCTCTCCTC | 108 | 90.60 |
| interferon regulatory factor 7 (*irf7*) | DN6933_c0_g1_i2 | F: GGCTCATAGAGCAGGTGGAG<br>R: CTGTCTTCGTCGTTGCAGTC | 115 | 81.89 |
| HLA class II histocompatibility antigen gamma chain (*cd74*) | DN13708_c0_g1_i6 | F: ACGCCAAGACACCTCTGACT<br>R: GGAAGGTCTCGTTGAACTGC | 108 | 96.45 |
| serum amyloid A 5 (*saa5*) | DN41536_c0_g1_i1 | F: AGAGTGGGTGCAGGAAAGAA<br>R: GAAGTCCTGGTGGCCTGTAA | 116 | 95.9 |
| T-cell surface glycoprotein CD4_a (*cd4a*)[c] | DN9678_c0_g2_i9 | F: CGTTAAGGTGCTGCAGATCA<br>R: GCGGAAACCATTTCAGTTGT | 122 | 84.85 |
| T-cell surface glycoprotein CD4_b (*cd4b*)[c] | DN24146_c0_g1_i7 | F: TGTGGGGTTAGCTCCTTCAC<br>R: TGTTTGCGATCTCACCTTTG | 138 | 94.24 |
| interleukin 1 beta (*il1b*)[c] | DN22448_c0_g2_i1 | F: ATTGTGTTCGAGCTCGGTTC<br>R: CGAACTATGGTCCGCTTCTC | 98 | 97.37 |
| toll-like receptor 5_a (*tlr5a*)[c] | DN29432_c0_g1_i1 | F: TGGACGAGTTTCAGCAGTTG<br>R: AGACCCCTCACATGTCCAAG | 129 | 95.58 |
| toll-like receptor 5_b (*tlr5b*)[c] | DN55824_c0_g1_i5 | F: CCATCATGCACTTTGTACGG<br>R: TGCTGTTGATCTCCCTGATG | 127 | 88.57 |
| tumour necrosis factor alpha (*tnfa*)[c] | DN26791_c0_g1_i1 | F: TTAGAAGGGAGCTGCGAAGA<br>R: ATGACGATCCGGTTGTTCTC | 119 | 90.06 |
| lymphocyte antigen 6 complex locus protein G6f (*ly6g6f*)[c] | DN12606_c0_g1_i8 | F: TCCATGTGGACGTGACTGTT<br>R: AACGGTGTCTGAGCCTGAGT | 100 | 88.17 |
| T-cell surface glycoprotein CD8 alpha chain (*cd8a*)[c] | DN11791_c0_g1_i1 | F: GCTTTGCTCTCTGGGCATAC<br>R: TCCGGGTTCTTAAGTGGTTG | 104 | 89.62 |
| immunoglobulin mu heavy chain_a (*ighma*)[c] | DN121_c0_g3_i3 | F: CAGCTTCTGGATTAGACTTTGA<br>R: GATGTTGTTACTGTTGTGTTGG | 107 | 90.17 |
| immunoglobulin mu heavy chain_c (*ighmc*)[c] | DN121_c0_g3_i4 | F: CAACATCCGGAATCACATTCAG<br>R: GATTTTGAGGTCCCACTACCAT | 112 | 87.68 |

| | | | | |
|---|---|---|---|---|
| interferon gamma (*infg*)[c] | DN81754_c0_g1_i1 | F: CTCTGGCTGGTTGTCTGTCA<br>R: TCGCTCTCTCGATGGAATCT | 105 | 90.75 |
| immunoglobulin delta heavy chain (*ighd*)[c] | DN1665_c0_g2_i7 | F: GGAGACAGTGTTGTGCTGGA<br>R: GGGCTTCAGGAAATTCAACA | 121 | 88.41 |
| toll-like receptor 7 (*tlr7*)[c] | DN760_c1_g2_i1 | F: GGCAAACTGGAAGAATTGGA<br>R: GAAGGGATTTGAGGGAGGAG | 100 | 90.55 |
| radical S-adenosyl methionine domain containing protein 2 / viperin (*rsad2*)[c] | DN16769_c0_g1_i1 | F: AGGAGAGGGTGAAGGGAGAG<br>R: ATCCAGAGGCAGGACAAATG | 133 | 98.47 |
| **Normaliser**[b] | | | | |
| eukaryotic translation initiation factor 3 subunit D (*etif3d*) | DN7623_c0_g1_i5 | F: AGCCAGATCAACCTGAGCAT<br>R: AGGCTGTACACCCGAATCAC | 134 | 86.49 |
| 60S ribosomal protein L32 (*rpl32*) | DN3569_c0_g1_i2 | F: GTAAGCCCAGGGGTATCGAC<br>R: GGGCAGCATGTACTTGGTCT | 107 | 80.08 |
| elongation factor 1 alpha_a (*ef1a_a*) | DN12280_c0_g1_i3 | F: CAAGGGATGGAAGATTGAGC<br>R: TGTTCCGATACCTCCGATTT | 151 | 83.81 |
| elongation factor 1 alpha_c (*ef1a_c*) | DN12280_c0_g1_i4 | F: AAGCGCTTTGAGGAAATCACC<br>R: GCTCGACCTTCCAACTCTTG | 160 | *95.60 |
| polyadenylate-binding protein 1_a (*pabpc1_a*) | DN6565_c0_g2_i3, DN6565_c0_g2_i4 | F: CAAGAACTTTGGGGAGGACA<br>R: TGACAAAGCCAAATCCCTTC | 125 | 84.76 |
| polyadenylate-binding protein 1_b (*pabpc1_b*) | DN6565_c0_g2_i5 | F: GACTCAGGAGGCAGCTGAAC<br>R: TCGCGCTCTTTACGAGATTT | 102 | 88.11 |

*4-pt standard curve

[a]Expression levels of the transcripts of interest were normalized to expression levels of these two transcripts.

[b]Candidate endogenous control transcripts

[c]Expression levels of these transcripts were low in lumpfish larvae; efficiencies are those reported for lumpfish head kidney (Gnanagobal et al., submitted).
